# WHO Regional Office for Europe’s Natural Experiment Studies Project: an introduction to the series

**DOI:** 10.1093/eurpub/cky195

**Published:** 2018-10-29

**Authors:** Andrew Snell, Aaron Reeves, Matthias Rieger, Gauden Galea, Kristina Mauer-Stender, Bente Mikkelsen, David Stuckler

**Affiliations:** 1 World Health Organisation Regional Office for Europe, Copenhagen, Denmark; 2 Department of Social Policy and Intervention, University of Oxford, Oxford, UK; 3 International Institute of Social Studies, Erasmus University Rotterdam, The Hague, The Netherlands; 4 World Health Organisation Country Office, Beijing, China; 5 Department of Social and Political Sciences, Università Bocconi, Milan, Italy

## The rationale

Fiscal, legislative and regulatory interventions now comprise the main WHO recommended ‘best-buys’ for preventing and controlling non-communicable diseases (NCDs). Yet these interventions are not well-suited to rigorous study using clinical trials, as they are often not feasible or ethical outside of smaller pilot studies. Currently we estimate that <1 out of every 10 NCD interventions are subject to real-world evaluation, including actions to ban trans-fat, tobacco control measures in the Framework Convention on Tobacco Control, alcohol minimum pricing and sugar-sweetened beverage taxes.^1^ This lack of impact evaluation obviates learning from what works, where and why. Without evidence it may also render these programmes more vulnerable to challenges from vested interests who seek to oppose them.

To begin addressing these challenges and improving the science of implementation,^2^^,^^3^ recent epidemiological advances have started to take advantage of the opportunity to evaluate policy implementation as a so-called ‘natural experiment’. Here, what differs from a randomized trial is that the intervention is not within the control of the research team but can be evaluated ‘as if’ it were an experiment. Importantly, this pays critical attention to developing a ‘control group’, which was not exposed to the intervention but is otherwise similar. A suite of methods have been developed to enable researchers and public health professionals to build context-appropriate evidence-based on rigorous designs regarding the impact of policies attempting to address NCDs, including regression adjustment, propensity scores, difference-in-differences, interrupted time series and synthetic controls.^4^ In light of these methodological developments, the UK Medical Research Council now recommends natural experiment designs for evaluating population health interventions.^4^

Many examples of natural experiments now exist in the scientific literature, from evaluating the impact of free-trade agreements on sugar-sweetened beverage consumption,^5^ to the impact of reducing housing benefit on mental health.^6^ Yet a gap remains in the uptake of these methods by practitioners working in health ministries. Often the methods employed in these settings involve simple descriptive statistics without giving careful consideration to techniques that can strengthen evaluation, such as constructing a control group or a clear ‘counterfactual’, identifying what would have happened absent the intervention.

To address this gap in real-world practice, we launched the WHO Regional Office for Europe’s (WHO/Europe) Natural Experiment Studies Project—an exercise in deploying these methods to begin the process of building a more influential evidence-base for the control of NCDs. This series presents four studies that were produced by national research teams as a result of the first iteration of this exercise.

### WHO natural experiment course in Copenhagen

In March 2017, WHO/Europe invited the Ministries of Health from eight countries from across the WHO European Region^7^—Austria, Finland, Hungary, Norway, Romania, Russian Federation, Turkey and Ukraine—to each nominate a public health research team (of two or three people) to attend a workshop at the WHO Regional Office in Copenhagen. To try and ensure the overall process would have both academic rigour and public health relevance we asked that the teams be made of a mix of health policy and academic experts. There were 20 participants in all.

The workshop presented the principles and application of natural experiment study techniques and was facilitated by David Stuckler and Aaron Reeves, and supported by external experts—Matthias Rieger, Galina Sakharova, Konstantin Vyshinskiy and Andrew Snell—and by experts from WHO/Europe. To participate, we invited research teams to identify a priority national population health intervention for NCD control and potential sources of data, and to commit to undertaking a natural experiment study. By the end of the two day workshop, the eight teams had developed a study design to proceed with.

During the following 14 months we provided support to the country teams, facilitating the design, data collection, analysis and drafting and editing of manuscripts. We also offered up to US$5000 of funding per team as needed, to get the study off the ground and for a suitable data analysis and statistical software licence. As had been anticipated from the outset, some teams were unable to progress to a final study. This happened for a variety of reasons:

- The process depended on collaboration within the team and with the experts, so any tension in priorities made it hard to proceed—in some cases studies were being dictated by the agenda of a short-term policy priority even if the policy was not amenable to evaluation using these methodologies;
- The teams were quite small, so there was little resilience to any reduction in capacity—changes or restructures in the ministries and in the academic institutions of some of the team members resulted in conflicting time-demands;
- The study methodologies being presented were very new to some research teams, and so any barriers to learning these made progress with remote support more difficult—barriers included conflicting time-demands, language and the baseline expertise of team members;
- Whilst the methods presented use mainly secondary data, there still needs to be a certain quality and detail in the data to make the analysis worthwhile—many of the studies suffered from a lack of consistent data covering a long enough time period, a lack of data on potential confounders and difficulty getting adequate data from comparator countries.

One recurring challenge facing the teams was the ability to identify a suitable ‘control group’. This marked a major shift in prior thinking among participants, where most population health analyses undertaken did not have a comparison group. A second recurring challenge was identifying data sources which track both intervention and control groups, as well as detail causal mechanisms involved.

Of the original eight countries that attended the March 2017 workshop, five countries completed a total of six studies. Two studies were excluded from peer-review, rejected by the series editors. Mainly these failed to go beyond traditional descriptive analyses. Four studies were sent to external peer-review with a recommendation to publish in this series, with the understanding that they were part of a pragmatic approach to building capacity on natural experiments.

### Natural experiment studies

Originally, the proposed natural experiment studies included alcohol, tobacco and nutrition interventions, but some of these could not be developed due to inadequate data.

Three of the studies that were completed in this iteration focussed on tobacco and one on trans-fats. Together they highlighted both the promise and the pitfalls of implementing natural experiment designs within a ministry of health:

- Austria examined the impact of a trans-fat ban introduced in 2009 on cardiovascular disease outcomes. To create a comparison group, it employed a synthetic control method, which creates an ‘artificial Austria’ based on data from otherwise similar OECD countries. This replicated and extended the design of an earlier natural experiment in Denmark.^8^ It found no clear evidence that the 2009 legislation improved CVD outcomes. However, several limitations make it difficult to draw firm conclusions, including a synthetic control that was poorly matched by characteristics for which there was no data, and gaps in the data during the period studied making a detailed time-series analysis difficult. In the short–term, it may be challenging to attribute a health outcome (such as CVD deaths) to the natural experiment. This remains a useful paper that might point towards the need for both incorporating long-term evaluations into such interventions and ensuring there is a multi-faceted and whole system approach to national CVD prevention and wider NCD control.
- Romania evaluated two consecutive sharp increases in tobacco taxation in 2009 (by 28%) and in 2010 (by 16%) on potential tobacco smuggling, hypothesizing that those regions bordering on other countries might have greater risks from illicit tobacco trade, so mitigating the health benefit of tax rises. This argument is often used in tobacco-funded research to undermine the case for taxes on tobacco consumption. Across the whole country, deaths from smoking attributable diseases declined over the study period, with steeper declines around the two years of tax hikes. Importantly, and in contrast to the fears noted above, there was no significant variation between regions within Romania. The study did have several limitations: the study period is short (2009–15) and this prohibits a rigorous pre-intervention trend analysis; and the study is unable to account for the varied and asymmetrical relationships between tax increases, the impact on smoking prevalence, and the impact on smoking-related hospitalizations, which are hard to account for when using ecological data. However, this is a timely and important paper that contributes to the evidence for national governments to support tobacco taxes and stand firm against tobacco counterarguments of illicit trade.
- The Russian team examined how Russia’s 2013 comprehensive Tobacco Control Law altered cardiovascular morbidity and mortality using a synthetic control design. They find evidence that hospital discharges for acute circulatory diseases were lower than expected after the reform, implying an associated benefit, but the impact on mortality rates from circulatory diseases was less clear. Again, residual confounders may explain the decline in the outcome because hospital discharges are not solely driven by tobacco. These make attributing the beneficial findings directly to the legislation difficult. However, there is a clear positive association and this study exploits an unusual opportunity in the evaluation of NCD control because Russia’s Tobacco Control Law presents a comprehensive, large scale and discrete intervention, rather than the iterative approach often taken in other settings. This is a useful contribution to the body of natural experiment studies for the evaluation of NCDs and takes another step towards demonstrating the beneficial impact of comprehensive tobacco control.
- The Turkey team conducted a time-series analysis spanning the period 1960–2016, measuring cigarette consumption. They estimate whether the trend in consumption changes dramatically after key policy reforms and supplement this with an analysis of the political discourse surrounding these reforms. Tobacco consumption increased with the entrance of multinational companies in Turkey but fell after the introduction of a national tobacco law in 1996. Policy discourse clearly shifted over this period, demonstrating a change in the political will to control tobacco consumption. However, the study does not fully unpack the causal mechanisms between policy change and the crude tobacco consumption trends. While the discourse analysis is an illuminating addition to the tobacco trend analysis, it is limited to only official notes from the legislature, rather than looking as well at other media. Despite these remaining concerns, the study presents an elegant and innovative use of two methodologies, and demonstrates some of the political motivations and priorities influencing effective tobacco control. This approach contributes to the natural experiment literature and offers a good approach to contextualizing natural experiment studies.

### The learning

Looking ahead, we learnt three major lessons for improving evaluations of public health interventions:

- Team structure: Most teams comprised a policy lead, a statistician, and a report writer. In most cases, it was simply asking too much in the time allocated to the project for policy leads to become familiar with the types of statistical analysis necessary to implement most natural experiment methods. Writing up the research is also a time-consuming process and other responsibilities often encroached on the project, making it difficult to find dedicated time to turn preliminary results into a finished article.
- Review and shared learning: A more formal inter-team review process might have strengthened the capacity building and shared learning throughout the project. Asking teams to review and comment on the work of others would have provided a unique insight into how each team was tackling challenges, which in many cases were similar. This would have allowed participants to learn from the strengths and limitations in the work of others to inform their own work.
- Earlier identification of data: We began this iteration of the project with a workshop on natural experiment methods in which we outlined the main research designs and helped teams develop study designs. However, this then became largely divorced from questions regarding the suitability of available data. We lost momentum in the project because it quickly became clear that some of the proposed designs were not feasible because of available and appropriate data. Remote contact in the first instance may have allowed the teams to identify more feasible natural experiments and scope appropriate data before attending the more intensive face-to-face workshop focussed on analyzing the data.

An ideal next step in this process would be to support countries to start the design stage of a natural experiment study ahead of the implementation of the public health intervention. This has been achieved by CEDAR in the case of the UK sugary drinks tax levy.^9^ However, this approach will be difficult especially in the context of some of the countries where capacity building is most important. In which case, addressing these three major lessons for future iterations of the exercise would be a priority.

## Conclusion

Natural experiment study designs are increasingly employed in academic research to shed light on how policy interventions affect health, but remain much less common among practitioners of various kinds, including within health ministries. We argue that, given our experience, some level of natural experiment evidence should be mandatory in NCD prevention interventions.

Our initiative shows the potential and promise of a straightforward programme to upgrade the quality of evidence and evaluation routinely collected at health ministries across the European Region.

There are several directions for future capacity-building programmes. One is to look to the future. Rather than retrospectively design studies, after the policies have been implemented, is to work closely with policymakers during the planning and roll-out. In this way, through minor tweaks in the policy or programme, policymakers and public health practitioners can better learn in real-time the live impact and identify gaps in successful implementation. Policymakers could, e.g. stagger the roll-out of the intervention so that some areas of the country receive it before others.

Another is to begin to think of all policies as real-world natural experiments which can be evaluated. This involves a critical step for those in health ministries to collect data on potential comparison or control groups. This provides critical evidence on the science of implementation. For example, examining whether trans-fat bans in Austria have the same impact on health as bans on trans-fat elsewhere may help reveal gaps in implementation or other real-world conditions that modify the policies’ success or failure. Similarly, replicating whether tobacco control in Russia delivers improvements in health creates the evidence-base necessary to protect health gains from future efforts that may seek to weaken tobacco control. The last few years have seen social security systems scaled back in many countries and a more robust evidence base on the health effects of welfare retrenchment may strengthen the case to protect social protection systems.

Finally, it is important to acknowledge the limitations of these studies, as with all observational designs. Evaluations in specific settings may not be generalizable and be contingent on factors that are assumed rather than explicitly modelled. A focus on endpoint health and disease outcomes may not always be possible, and proximal impacts or implementation indicators may better serve to assess the value of natural experiments. The best natural experiments often involve multiple methods and acknowledge that policies tend to have nuances in implementation across all locations, despite what the formal expectations may have been. Qualitative or ethnographic approaches combined with process tracing can provide much needed insight into how policies have been implemented and whether the experience on the ground may partially explain outcomes. Where it is not possible to find an appropriate control group, there is still an important role for more traditional econometric or epidemiologic methods.

The public health community has long committed itself to deepen the understanding of what works, where and why. Natural experiment study designs are a powerful tool to do so. Extending it to public health settings across the WHO European Region, building on the insights from our first attempt to do so here, is a good place to start.
